# Extract from Paper by Wm. Porter, M.D., on Nasal Catarrh

**Published:** 1876-04

**Authors:** 


### EXTRACT FROM PAPER BY WM. PORTER, M. D., ON NASAL CATARRH.

The symptoms of chronic catarrh are only too apparent. The heat and irritation of acute inflammation may be wanting, but instead, we have fullness, and a sense of weight about the frontal sinuses, difficulty of nasal respiration, and the troublesome secretion already described. Part of this secretion comes away through the nostril, but a large quantity of it passes down the pharyngeal wall, whence it is expectorated, or passes into the stomach. If the latter, it may set up inflammation, or at least irritation in the intestinal tract. From this, doubtless, come many dyspeptic symptoms. If ulceration has taken place there is a fetor preceptible with every expiration, and a constant, not always copious, discharge, sometimes mixed with blood.

In the treatment of this obstinate affection there are four main points to be considered. The *first* has reference to the predisposing cause, the constitutional fault which must be rectified. In the strumous type, iodide of iron, or iodiform and iron, with cod liver oil, is generally indicated. The treatment of catarrh from syphilis is obvious. If there is ulceration, iodide of potassium with ammonia, and some form of tonic, are called for; but, if no ulcers exist, the bichloride of mercury in small doses, if persevered with, has no equal. These cases are much more manageable than those of scrofulous origin.

In the catarrhal diathesis, which we have noted, the majority of cases seem to do well with some preparation of phosphorus. This, with the addition of nux vomica, has been useful in relieving obstinate constipation and nervous lassitude in patients of this kind.

In all these conditions, good food, rest and regular habits of life must be enjoined. The social enjoyment and happiness of the patient should not be neglected, but if possible improved knowing, as physicians must, how great is the influence of the mind upon the nervous system, and of the nervous upon the nutrient.

*Secondly.*—The local cause of catarrh must be removed. If there is a polyp it should be enclosed in a snare, or seized with the forceps, and be brought out. In the glandular hypertrophy mentioned, by aid of the rynoscope, the part may be touched with nitrate of silver, and the more pendulous portions destroyed, while all that would irritate the nasal mucous membrane must be avoided or guarded against. But this alone will not suffice. The membrane is already hypertrophied, and the secretion abnormal.

A *third* important item is to keep the part thoroughly cleansed, so as to remove all the adherent mucous and incrustations. Nothing does this better than a weak solution of common salt in tepid water. It is not enough that it should be used through the nasal douche. (The writer cannot afford to ignore the remonstrances of Drs. Roosa, Spencer, and others, against the use of the nasal douche, in that the fluid. by passing along the Eustachien tube, may do injury in the tympanic cavity. He would, therefore, urge that it never be used by the patient, except under the immediate supervision of the physician, who should be not merely an *instructor* but the *operator;* that the solution should never exceed a drachm of salt to the pint of water; that the salt should be well dissolved; and that the pressure should not exceed that of a column of water of twelve inches. These requirements, if carefully attended to, will obviate this danger. That so few cases have been recorded in which injury from the use of the douche has occurred, among the thousands who daily abuse it, sufficiently attests its harmlessness if properly applied.) This means, while valuable, does not reach the upper, and sometimes most important part of the cavity. An easy way of affecting this is to attach to the douche a tube with an aperture on one side, which may be passed into the nostril, through which a stream of the solution may be directed upward against the part, or the tube may be attached to a nasal syringe. Dr. Rumbold recommends a cathether with numerous small apertures, through which a spray of air and water is sent with good effect. If the disease has become ulcerative, a deodorizing solution of permanganate of potassium or salicylic acid may be used in a spray, after the cleansing.

When this is done, much still remains. *Fourthly*, and last, but most important, is the local medication. If there are ulcers, they may be touched with iodine in glycerine and water, with the addition of a little iodide of potassium, or with weak solutions of nitrate of silver. The latter is most useful where there is thickening of the membrane. Hydrate of chloral answers a good purpose, where the ulceration is sluggish, if directly applied. It may be used in from five to fifteen grains to the ounce of the menstruum. Where the thickening is not marked iodide vapor does well. A few drops of a concentrated solution of iodine, containing a little conium or lupulin, may be placed in a Roosa's inhaler, and the vapor propelled by means of the double bulbs or a small bellows. This should be used for at least fifteen minutes each day. After a few applications of this vapor, a free flow of serun is induced, which lessens the infiltration and its attendant symptoms. If there is deafness from narrowing of the Eustachian tube, it may sometimes be much relieved if the vapor is passed along it by means of inflation by the Valsalvian method.

In many cases, the frequent use of a snuff composed of camphor, tannic and salicylic acid, is advantageous.

It is not the intention of the writer to publish a routine treatment in calling attention to these points. In no disease is it more necessary to individualize each case, and to watch and treat each symptom that may arise. Not only this, but in each case determined perseverance is required on the part both of physician and patient. A condition that has existed for years cannot be removed in a day. By means of the improved facilities for examining and treating the subject of this disease, catarrh is rendered manageable—nay, more, it is in many cases, curable; an assertion which the testimony of many who are earnestly working in this direction goes far to prove. In closing, it may not be improper to add that our experience has justified the endorsement of this treatment, and to express the hope that it may be found useful to others.—*Western Lancet.*

